# Ethnobotanical survey on plants used in the treatment of candidiasis in traditional markets of southern Benin

**DOI:** 10.1186/s12906-020-03080-6

**Published:** 2020-09-21

**Authors:** Brice Armand Fanou, Jean Robert Klotoe, Lauris Fah, Victorien Dougnon, Charles Hornel Koudokpon, Ghislaine Toko, Frédéric Loko

**Affiliations:** 1 Unité de Recherche en Microbiologie Appliquée et Pharmacologie des substances naturelles (URMAPha), Laboratoire de Recherche en Biologie Appliquée (LARBA), Ecole Polytechnique d’Abomey-Calavi (EPAC), Université d’Abomey-Calavi, 01BP2009 Cotonou, Bénin; 2 Ecole Normale Supérieure de Natitingou, Université Nationale des Sciences, Technologie, Ingénierie et Mathématiques, BP72 Natitingou, Benin; 3 Centre de Recherche Enthomologique de Cotonou (CREC), Cotonou, Benin

**Keywords:** Candidiasis, Ethnobotanical survey, Medicinal plants, Southern Benin

## Abstract

**Background:**

Candidiasis, an opportunistic cosmopolitan disease is nowadays like bacterial infections which is a real public health problem. In view of the emergence of Candida strains resistant to existing antifungal agents, alternative solutions should be considered. This is the purpose of this ethnobotanical survey, which aims to identify the medicinal plant species traditionally used to treat candidiasis in traditional markets of southern Benin.

**Methods:**

The study was performed from October 2015 to January 2018 in the traditional markets of Southern-Benin. Data were collected by two complementary methods: triplet purchase of medicinal recipes (ATRM) from herbalists markets and semi-structured interview (ISS) from traditional healers.

**Results:**

A total of 109 species of medicinal plants belonging to 44 families have been listed and identified. The most frequently cited species were *Pteleopsis suberosa* Engl. & Diels, *Lantana camara* L., *Cyanthillium cinereum* (L.) H. Rob, *Ocimum gratissimum* L. and *Lippia multiflora* Moldenke with respectively 43.84, 39.73 and 34.25% citation frequencies for the last three species respectively. Leguminosae (20.18%), Euphorbiaceae (5.50%) and Apocynaceae (5.50%) were the most represented botanical families. Leafy stems were more used than other plant organs. The decoction and the oral route were the most appropriate methods of preparation and administration reported by traditional healers.

**Conclusion:**

Benin’s plant cover is made up of a wide variety of medicinal plant species used in the traditionnal treatment of candidiasis and which may constitute new sources of medicines to be developed.

## Background

Candidiasis is a cosmopolitan fungal infection associated with yeasts of the genus Candida. It is one of the most common opportunistic infections in tropical areas, with a frequency ranging from 33 to 47% in [1]. In recent decades, their prevalence has been steadily increasing, especially among patients in intensive care and with the advent of HIV/AIDS infection [1–3]. They affect all types of tissues and mainly mucous membranes [4–6]. It is reported that 25% of urinary tract infections were related to Candida spp. [7]. 50 to 75% of women of childbearing age developed vulvovaginal candidiasis annually and 5 to 8%, or about 75 million women, can be affected at least four times in a year [8–10]. Oral infections are common but are found in children and immune compromised people [11]. Ten million cases of oral candidiasis and 2 million esophageal candidiasis are reported annually in people living with HIV. Most seriously, invasive fungal infections are reported to kill more than tuberculosis and malaria and in 90% of fungal deaths, candidiasis ranks second behind cryptococcosis and ahead of aspergillosis and pneumocystis [12–14]. They are fatal in 40% of hospital sepsis cases according to letter No. 72 from the Pasteur Institute [15] and are the leading nosocomial fungal disease [9, 16, 17]. Their therapeutic management requires the use of antifungals to which *Candida spp.* strains are increasingly resistant [7, 18, 19]. Indeed, high levels of *Candida spp*. resistance to fluconazole have been identified in several countries [20–22]. Candida spp. resistance even extends to amphotericin B [23], one of the last used antifungals [24, 25]. This emergence of strains resistant to common molecules is, like the emergence of bacterial resistance to antibiotics, of a great public health concern for which sustainable alternative solutions must be found very quickly. In African countries, these alternative solutions involve the use of medicinal plants. Indeed, the use of plants to treat diseases is an old practice [26–29] and endogenous to populations. According to several authors, in Africa and Asia, 80% of the population continues to use traditional medicines rather than the so-called modern medicines for primary health care for various reasons [30–32]. Today, with the support of WHO [33], many research focuses on plants to look for active compounds [3, 34–36]. In this sense, some research carried out on plants has shown in vitro their antifungal potential [37–40] and could thus constitute new sources of bioactive molecules [31, 41].

Unfortunately, plant resources are under significant anthropogenic pressure which dangerously reduces plant biodiversity. According to Djégo et al., Benin loses 60,000 ha of forest per year, an annual rate of deforestation estimated at 1.2%. This deforestation is not without consequences for conservation on medicinal plants. Several medicinal species have thus disappeared or are threatened with extinction. It is therefore important to ensure their conservation for the next generations. This requires their knowledge and compliance with the rules of sustainable use [42].

In Benin, the plant species indicated for candidiasis treatment is still poorly known, because few studies have been carried out on the antifungal properties of some medicinal plants [43]. This work is therefore the first on aiming to identify the medicinal plants indicated by herbalists and traditional healers in southern Benin for the treatment of candidiasis.

## Methods

### Framework

**Fig. 1:**
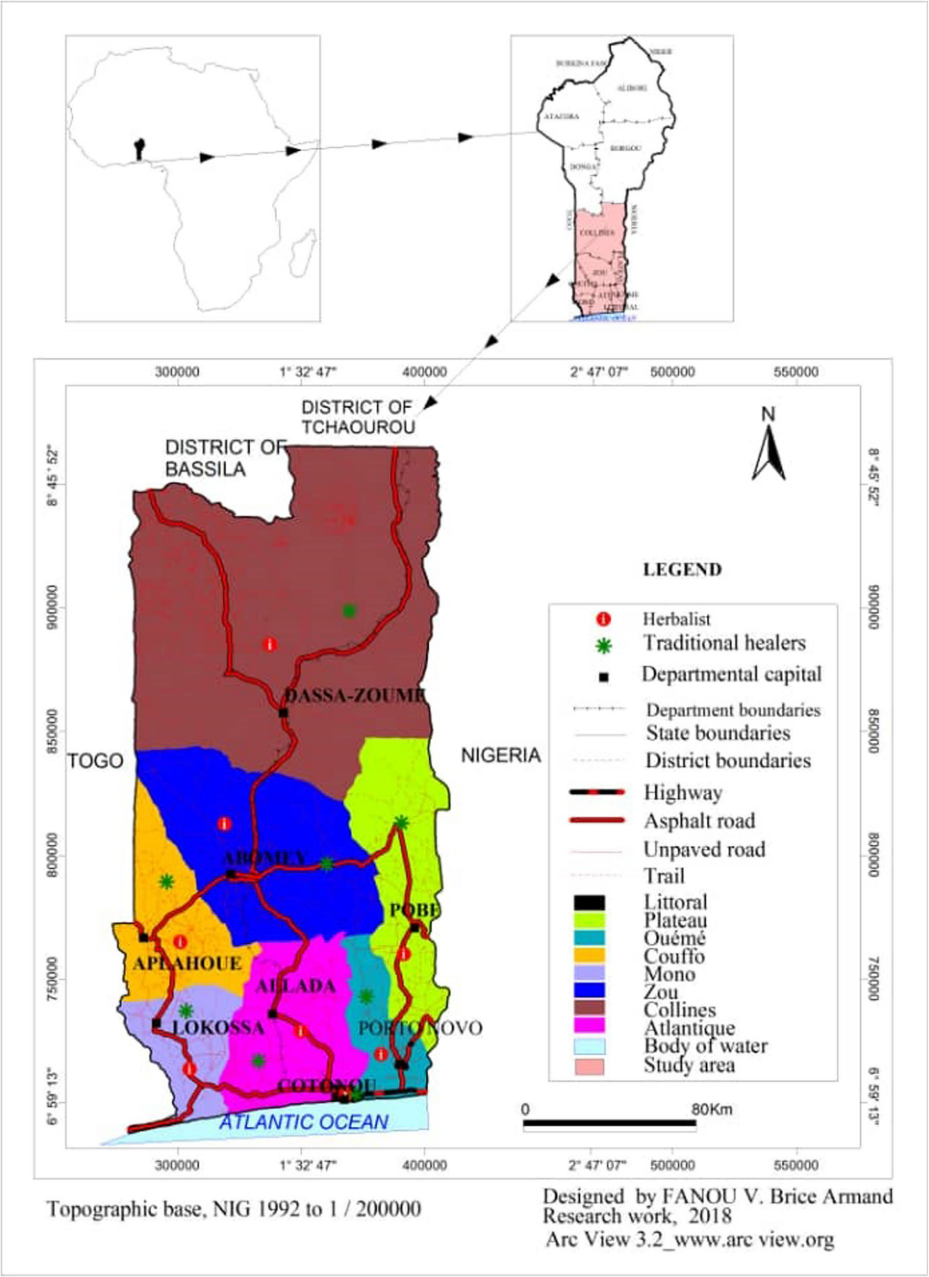
Location of traditional markets covered by the ethnobotanical survey

The surveys were conducted in Benin from October 2015 to January 2017, in the geographical area between longitude 03° 40′ and 04° 11′ north and the 09° 16′ - 09° 52′ east meridians. It is bordered to the north by Bassila and Tchaourou municipalities (departments of Borgou and Donga), to the west by Togo, to the east by Nigeria and to the south by the Atlantic Ocean (Fig. 1). It covers eight of the twelve departments in the Republic of Benin and corresponds to thirty-six (36) municipalities out of the forty-one in the southern part of Benin.

The localities concerned by study are:

- Cotonou (09 traditional markets: Agla, Akpakpa, Dantokpa, Fifadji, Fidjrossè, Gbèdjromèdé, Gbogbanou, Mènontin, Wologuèdè)
- Abomey-Calavi (04 traditional markets: Atrokpocodji, Godomey, Tokan, Tokpa)
- Porto-Novo (02 traditional markets: Ahidahomè, Ouando)
- Abomey (01 traditional market: Houndjro)
- Adjara (01 traditional market: Adjarra)
- Azovè (01 traditional market: Azovèhi)
- Bohicon (01 traditional market: Gbohicon)
- Covè (01 traditional market: Covèhi)
- Dassa-Zoumè (01 traditional market: Dassa)
- Dogbo-Tota (01 traditional market: Dogbo)
- Klouékanmey (01 traditional market: Klouékanmè)
- Lokossa (01 traditional market: Lokossahimè)
- Ouidah (01 traditional market: Kpassè)
- Savalou (01 traditional market: Savalouhi)
- Savè (01 traditional market: Savè)
- Tori (01 traditional market: Gbodjè).

#### Equipment

Equipment used in this study consists mainly of survey sheets for information collection, a digital camera, a self-recording audio device for interview recording, a position marker (GPRS).

#### Methods

The surveys were conducted with two groups of professionals: market herbalists and traditional healers. Market herbalists sell herbal remedies and also compose recipes for the treatment of illnesses. They don’t directly treat patients. This role is reserved for traditional healers. The traditional healers interviewed are recognized by the National Program for the Promotion of Traditional Medicine in Benin. As for herbalists, they are registered with the market management company.

Two (02) methods were used for each group of informants. Thus, among market herbalists, the triplet purchase of medicinal recipes (ATRM) method was used and the semi-structured interview method (ISS) was used among traditional healers [44–47].

Interviews were conducted in three local languages (Fon, Goun or Mina) and then in French for those who were literate. An interpreter was recruited from each location where local language spoken was not understand.

Data collected consisted of the socio-demographic characteristics of the respondents (sex, age, professional experience, mode of entry into the profession) and information on the recipes used to treat candidiasis (composition of recipes, local names and parts of plants used, methods of preparation and administration routes of the recipes served, dosage, bans and side effects). The study focused on cutaneous candidiasis “Atita”; oral candidiasis “*Noumè vo*”, genital candidiasis or vulvovaginitis “*Atita do Gnonnnou houé*”. Pictures of the recipes and plants mentioned were taken and sampled. Herbariums were then created for taxonomic identification (scientific name, family).

#### Identification of plant species

The species mentioned by the markets herbalist were purchased and those indicated by the traditional healers were harvested. Each time, care was taken to collect or purchase fresh samples for identification. These collected samples were identified at the National Herbarium of Benin of the University of Abomey-Calavi (UAC-Benin) using the analytical flora of Benin by Akoègninou et al. [48]. The botanical nomenclature used is that of the “The Plant List” database available on the website www.theplantlist.org.

Listed plant species were checked against the IUCN Red List Categories (Critically Endangered, Endangered, Vulnerable, Near Threatened) to identify endangered species.

#### Data analysis

The data collected were processed using Microsoft Excel version 2010 software, which was also used to draw graphs (Pie charts, charts and histograms). The variables were presented in percentage.

The phototherapeutic importance of each species was assessed by calculating four parameters, namely:

- The informant fidelity index (**FI**)

$FI=\frac{\mathrm{Nc}}{\mathrm{Nt}}x100$ (Nc = number of informants in a given category who cited the species; Nt = total number of informants in all categories who cited the same species).

It makes it possible to assess the relationship between a given plant species and its use by herbalists and/or traditional healers in the treatment of candidiasis [49, 50].

- The Informant Consensus Factor (**ICF**) calculated by the formula (total number of revenues minus total number of informants / total number of revenues minus 1) [49, 51, 52]. This consensus factor of informants here expresses their “approval rate” in related to the plants used to make the recipes for candidiasis treatment. The value of the ICF is less than or equal to 1, so when the value of the ICF is less than 0.5, the consensus is low, when it is between 0.5 and 0.75 the consensus is high and very high when the ICF tends towards 1.
- The citation frequency (**Fc**) expressed as a percentage (%) and obtained by the formula: (number of citations of a species / total number of citations of species) × 100 [53, 54].
- The contribution of each plant species to recipes composition (**Cpr**) expressed as a percentage (%). It is also the frequency with which plants are involved in recipes. It was calculated by the formula (number of recipes using the plant species / total number of recipes) × 100 (55).

## Results

### Sociodemographic characteristics of responders

**Fig. 2:**
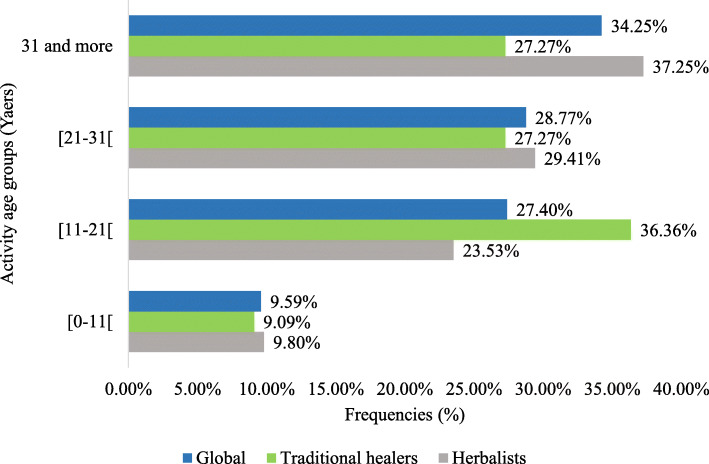
Professional experience of informants

**Fig. 3:**
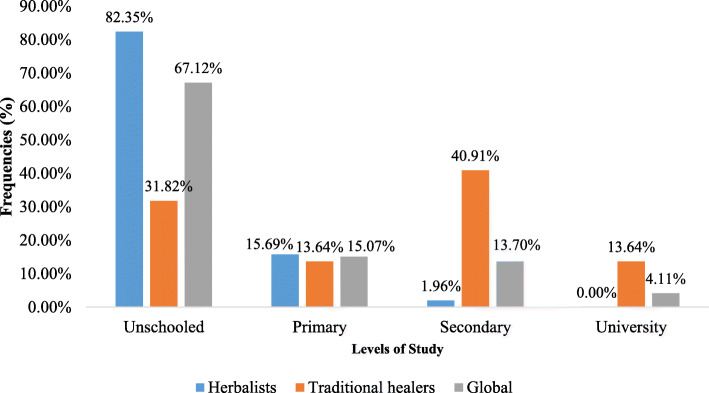
Educational level of respondents

The recipes were provided by seventy-three (73) informants, fifty-one (51) market herbalists and twenty-two (22) traditional healers with an average age of 52 ± 14.65 years. All the traditional healers were men and the market herbalists were women. The average ages of traditional healers and herbalists were 53.54 ± 14.79 years and 51.31 ± 14.63 years respectively, with 40 to 60 years as predominant age group (Table 1). The majority (90%) of informants were experienced in this activity for at least ten years. And more than half of them (63%) had more than twenty years’ experience (Fig. 2) but traditional healers seem to be less experienced than market herbalists. Two thirds (67%) of the responders were out of school. However, the traditional healers sub-group has more educated people than the market herbalists (Fig. 3).

**Table 1:** Age distribution of respondents

Age groups (years)	Herbalists n (Frequency in %)	Traditional healers n (Frequency in %)	Overall n (Frequency in %)
Below 20	0 (**0%**)	0 (0%)	0 (0%)
[20–40[	9 (**17.65%**)	3 (**13.64%**)	12 (**16.44%**)
[40–60[	26 (**50.98%**)	12 (**54.54%**)	38 (**52.05%**)
60 and more	16 (**31.37%**)	7 (**31.82%**)	23 (**31.51%**)
Total	51 (100.00%)	22 (100.00%)	73 (100%)

### Inventory of recipes and plant species

A total of 124 recipes were provided, 81 (65%) of which were from market herbalists. Table 2 gives the composition of the recipes served. The recipes are made up of a single plant species (21.77%) or a combination of plants species (78.23%). The number of plants per recipe varied according to the category of informants. It was obvious that the proportion of plants constituting the recipes for market herbalists was in opposite trend compared to that of traditional healers. Indeed, plant associations were much more noticeable among herbalists with 45.68% of recipes composed of more than 6 plant species compared to only 6.98% among traditional healers. It should be noted that in some cases (6.45%) non-plant elements such as mineral compound (kaolin, alum or salt) and sulphur are added to plant organs in the composition of recipes.

**Table 2:** Composition of recipes

	n (Frequency in %)					
	Single plants	Plants + NPE	1Plant	2plants	3 to 5plants	6plants and more
Herbalists	80 (98.77%)	1 (1.23%)	10 (12.35%)	11 (13.58%)	23 (28.40%)	37 (45.68%)
Traditional healers	36 (83.72%)	7 (16.28%)	17 (30.23%)	16 (37.21%)	7 (16.28%)	3 (6.98%)
**Global**	116 (93.55%)	8 (6.45%)	23 (21.77%)	27 (21.77%)	30 (24.19%)	40 (32.26%)

*NPE* non-plant elements (Kaolin, alum, sulphur)

The high proportion of recipes provided by herbalists could have a pecuniary cause. Indeed, since the recipes were bought from herbalists and the cost of a recipe varies most of the time between 300f CFA for the cheapest and 500f CFA or even 1200f CFA for the most expensive, the herbalists prefer to pay for the same affection all that they know as recipes.

**Fig. 4:**
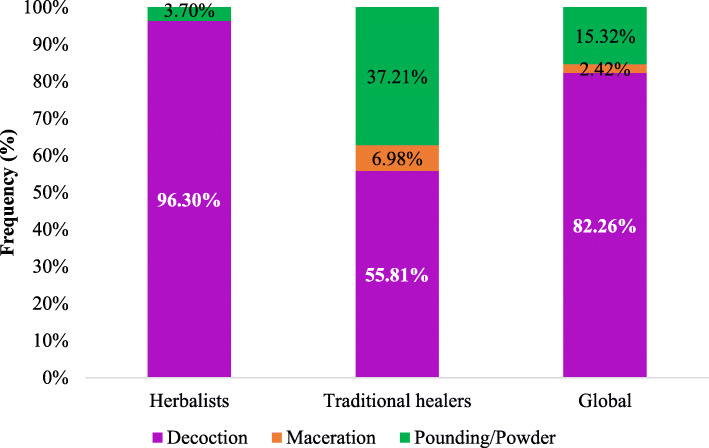
Recipe preparation methods

**Fig. 5:**
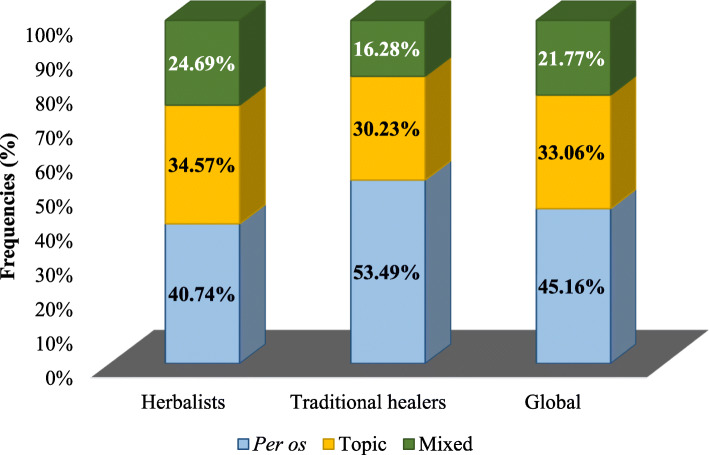
Administration modes of preparation

The most used preparation method is decoction (82%). Only traditional healers talked about maceration, corresponding to only 2.45% of recipe preparation methods (Fig. 4). The oral route was the most indicated route (45.16%) by informants for preparation administering (Fig. 5).

The floristic inventory of species identified 109 species of 101 genera and divided into 44 botanical families (Table 3) with an overall ICF consensus index of 0.12.

**Table 3:** List of plant species used for traditional treatment of candidiasis in traditional markets of southern Benin

N°	Voucher Number	Scientific names	Vernacular names	Botanical families	Parts of plants used	Mode of preparation	administration route	Fc (%)	Cpr (%)	IFh (%)	IFt (%)	Previous References
1	YH 337 / HNB	*Abutilon mauritianum* (Jacq.) Medik.	Adansounyi (f,g)	Malvaceae	Tf	Dec	Oral	0.92	0.81	100	0	
2	YH 358 / HNB	*Acacia nilotica* (L.) Delile	Bani (f, y)	Leguminosae	Fr	Dec	Oral + Topic	4.59	4.03	100	0	
3	AA 6752 / HNB	*Acalypha wilkesiana* Müll. Arg	“Flowa”	Euphorbiaceae	Tf	Dec	Oral + Topic	1.83	1.61	0	100	
4	YH 286 / HNB	*Aframomum melegueta* K. Schum.	Atakoun (f,g)	Zingiberaceae	Fr	R cend	Oral	1.83	1.61	50	50	
5	YH 338 / HNB	*Afzelia africana* Pers.	Kpakpatin / Kpakpa Jidé / Kpakpa Gidé (f)	Leguminosae	Tf	Powder	Oral + Topic	1.83	1.61	50	50	
6	YH 339 / HNB	*Allium cepa* L.	Ayoma, Ayomasa, vovo, massa (f, g)	Amaryllidaceae	Fr	Dec	Oral + Topic	2.75	2.42	100	0	
7	YH 287 / HNB	*Allium sativum* L.	Ayo (f, g)	Amaryllidaceae	Fr	Dec; Mac	Oral + Topic	1.83	1.61	0	100	[55]
8	YH 345 / HNB	*Amaranthus spinosus* L.	Handoukpo (f)	Amaranthaceae	Tf	Dec; Mac; Mettre en poudre	Oral	2.75	2.42	0	100	
9	YH 340 / HNB	*Anacardium occidentale* L.	Akaju (tin), Lakazu (f,g)	Anacardiaceae	B	Dec	Oral	3.67	3.23	100	0	
10	YH 341 / HNB	*Annona muricata* L.	Nyiglwe, anyiglwe (f); Chap-chap (fr)	Annonaceae	Tf	Dec	Oral + Topic	2.75	2.42	100	0	
11	YH 342 / HNB	*Anogeissus leiocarpa* (DC) Guill. & Perr.	Hlihon, hilihon (f)	Combretaceae	Tf	Dec	Oral + Topic	1.83	1.61	50	50	
12	YH 343 / HNB	*Antiaris toxicaria* Lesch.	Guxotin (t)	Moraceae	Le	Powder	Topic	0.92	0.81	0	100	
					S							
13	YH 395 / HNB	*Bambusa vulgaris* Schrad.	Bambou (fr)	Poaceae	Tf	Dec	Oral + Topic	9.17	8.06	100	0	
14	YH 400 / HNB	*Bauhinia reticulata* DC.	Kpakpa, Klon (f); Kongbo (g)	Leguminosae	Tf	Dec	Oral + Topic	0.92	0.81	0	100	
15	YH 344 / HNB	*Blighia sapida* K. D. Köening	Lisetin (f);	Sapindaceae	Fr	Powder	Topic	0.92	0.81	0	100	
16	YH 293 / HNB	*Bridelia ferruginea* Benth.	Honsukokwe, Hongla (f, g);	Phyllanthaceae	B	Dec	Oral + Topic	15.6	13.71	100	0	
					R							
17	YH 296 / HNB	*Caesalpinia bonduc* (L.) Roxb.	Ajikun, ajikwin (f, g)	Leguminosae	Tf	Dec; Pounding	Oral + Topic	3.67	3.23	25	75	
18	YH 347 / HNB	*Caesalpinia pulcherrima* (L.) Sw.	Orgeuil de Chine (fr);	Leguminosae	Tf	Dec	Oral	3.67	3.23	50	50	
19	YH 348 / HNB	*Cajanus cajan* (L.) Millsp.	Klwekun (f,g)	Leguminosae	Tf	Dec	Oral + Topic	3.67	3.23	100	0	
20	YH 298 / HNB	*Carica papaya* L.	Kpèn (tin) (l’arbre), Jikpèn (le fruit) (f);	Caricaceae	R	Powder	Topic	0.92	0.81	0	100	
21	YH 290 / HNB	*Carissa spinarum* L.	Ahanzo (f);	Apocynaceae	R	Dec	Oral + Topic	6.42	5.65	71	29	
					B							
22	YH 349 / HNB	*Catharanthus roseus* (L.) G. Don	Flawe (f)	Apocynaceae	Tf	Dec	Oral	0.92	0.81	0	100	[31]
23	YH 350 / HNB	*Ceropegia fusiformis* N.E.Br.	Zunkuju wewe (f)	Apocynaceae	R	Mac	Oral	0.92	0.81	100	0	
24	YH 303 / HNB	*Citrus aurantiifolia* (Christm.) Swingle	Klé, (f)	Rutaceae	Fr	Dec	Oral + Topic	2.75	2.42	33	67	
25	YH 353 / HNB	*Clausena anisata* (WilId.) Hook.f. ex Benth.	Gbozohouin/ Gbosu / Zohwɛn/ Gboma Duwa Zohwɛn (f)	Rutaceae	Le	Dec	Oral + Topic	6.42	5.65	86	14	
					R							
26	YH 354 / HNB	*Clerodendrum capitatum* (WilId.) Schumach. & Thonn.	Zoplotin (f); Wèma (f,g)	Lamiaceae	Tf	Dec	Oral + Topic	2.75	2.42	100	0	
27	YH 306 / HNB	*Cola nitida* (Vent.) Sebott & EndI.	Gbanja, Goro, Golo	Malvaceae	Fr	Dec	Oral	0.92	0.81	0	100	
28	YH 356 / HNB	*Combretum micranthum* G.Don	Vrai Kinkéliba (fr); kinikiniba (f)	Combretaceae	Tf	Dec	Oral	0.92	0.81	100	0	
29	YH 383 / HNB	*Combretum paniculatum* Vent.	Dongbo (Dokpo)	Combretaceae	Tf	Dec	Oral	0.92	0.81	0	100	
30	YH 357 / HNB	*Commiphora africana* (A.Rich.) Engl.	Liji (man)(f);	Burseraceae	Tf	Dec	Oral	0.92	0.81	0	100	
31	YH 359 / HNB	*Crateva adansonii* DC.	Onton zunzen; Wonton Zinzwen (f, g); Sharu wéwé (y)	Capparaceae	Tf	Dec	Oral + Topic	10.09	8.87	64	36	
32	YH 360 / HNB	*Croton gratissimus* Burch.	Jelele, jebele (f,g)	Euphorbiaceae	Tf	Dec	Oral + Topic	2.75	2.42	100	0	
33	YH 355 / HNB	*Croton lobatus* L.	Alòvi atòn (f)	Euphorbiaceae	Tf	Dec	Oral + Topic	1.83	1.61	100	0	
34	YH 361 / HNB	*Curculigo pilosa* (Schumach. & Thonn.) Engl.	Ayote, ayoglèn, (f)	Hypoxidaceae	Fr	Mac	Oral + Topic	0.92	0.81	100	0	
35	YH 419 / HNB	*Cyanthillium cinereum* (L.) H.Rob.	Hunsukusɛ / Hunsikonu (F)	Compositae	Pe	Dec; Pounding; Grilling	Oral + Topic	22.94	20.16	100	0	
36	YH 362 / HNB	*Cymbopogon citratus* (DC.) Stapf	Tcha; Ca / Ti (man) (f)	Poaceae	Le	Dec	Oral	2.75	2.42	100	0	[56]
37	YH 363 / HNB	*Daniellia oliveri* (Rolfe) Hutch. & Dalziel	Zaxaya (f)	Leguminosae	Tf	Dec	Oral + Topic	1.83	1.61	100	0	
					R							
38	YH 310 / HNB	*Desmodium velutinum* (Willd.) DC.	Tèd’avowu, Zɛn’ali (f)	Leguminosae	Tf	Dec	Oral	0.92	0.81	100	0	
39	YH 365 / HNB	*Detarium microcarpum* Guill. & Perr.	Dakpa, dagpa (f);	Leguminosae	R	Dec	Oral	0.92	0.81	100	0	
40	YH 366 / HNB	*Dialium guineense* WiIld.	Asònswèn, asiswetin, aswenswen (f, g);	Leguminosae	Tf	Dec	Oral	0.92	0.81	100	0	
41	YH 367 / HNB	*Dichapetalum madagascariense* Poir	Gbaglo (f)	Dichapetalaceae	Tf	Dec	Oral	0.92	0.81	100	0	
42	YH 368 / HNB	*Dichrostachys cinerea* (L.) Wight & Arn.	abadawèn, badawèn (f)	Leguminosae	Le	Dec	Oral	0.92	0.81	0	100	
43	YH 346 / HNB	*Diodella scandens* (Sw.) Bacigalupo & E.L.Cabral	Sèhoun (f)	Rubiaceae	Tf	Dec	Oral + Topic	1.83	1.61	100	0	
44	YH 351 / HNB	*Dysphania ambrosioides* (L.) Mosyakin & Clemants	Amatluzu, godo (f)	Amaranthaceae	Pe	Dec	Oral + Topic	4.59	4.03	100	0	
45	YH 369 / HNB	*Echinochloa pyramidalis* (Lam.) Hitchc. & Chase	Woko (g);	Poaceae	Tf	Dec	Oral + Topic	1.83	1.61	100	0	
46	YH 370 / HNB	*Ehretia cymosa* Thonn.	Zozoma (f); myoma (g);	Boraginaceae	Tf	Dec	Oral + Topic	5.5	4.84	100	0	
47	YH 371 / HNB	*Entada gigas* (L.) Fawc & Rendle	Gbagbakwin (f);	Leguminosae	L	Powder	Oral + Topic	1.83	1.61	50	50	
48	YH 372 / HNB	*Erythrina senegalensis* DC.	Kpaklesi, pakléwésè (f);	Leguminosae	B	Dec	Oral	0.92	0.81	100	0	
49	YH 373 / HNB	*Eucalyptus camaldulensis* Dehnh.	Eucalyptus rouge, eucalyptus rostré,	Myrtaceae	Tf	Dec	Topic	0.92	0.81	100	0	
50	YH 374 / HNB	*Euphorbia hirta* L.	Hundi hundi asu (f)	Euphorbiaceae	Tf	Dec	Oral	1.83	1.61	100	0	
51	YH 314 / HNB	*Flacourtia flavescens* Willd	Gbohunkaje / Gbowunkajɛ (f);	Salicaceae	Tf	Dec	Oral	0.92	0.81	100	0	
52	YH 260 / HNB	*Garcinia kola* Heckel	Ahowetin (l’arbre), ahowé (la graine) (f, g); arowé (f);	Clusiaceae	Fr	Dec	Oral + Topic	3.67	3.23	0	100	[57]
53	YH 352 / HNB	*Gladiolus dalenii* van Geel	Baka [petit oignon]	Iridaceae	Fr	Dec	Oral + Topic	3.67	3.23	50	50	
54	YH 376 / HNB	*Gossypium hirsutum* L.	Avokanfoun tin (f)	Malvaceae	Tf	Dec	Topic	2.75	2.42	100	0	
55	YH 418 / HNB	*Gymnanthemum coloratum* (Wild.) H.Rob. & B.Kahn	Alomaklu/ Amavive gbémenton (f)/ Gbélé man	Compositae	Pe	Dec	Topic	0.92	0.81	100	0	
56	YH 377 / HNB	*Heliotropium indicum* L.	Koklon son, Kokloden; koklosu denpaja (f)	Boraginaceae	Tf	Dec	Topic	0.92	0.81	100	0	
57	YH 420 / HNB	*Hymenocardia acida* Tul.	Mlanlanvê man (f); Manlanvi (g)	Phyllanthaceae	Tf	Pounding	Oral	0.92	0.81	100	0	
58	YH 378 / HNB	*Hyptis suaveolens* (L.) Poit.	Afio, Xweflu, hwéflou (f)	Lamiaceae	Pe	Dec	Oral + Topic	19.27	16.94	100	0	[30]
59	YH 421 / HNB	*Imperata cylindrica* (L.) Raeusch.	Xè, xètin (f), Oxɛ tin	Poaceae	R	Dec	Oral + Topic	5.5	4.84	100	0	
					Tf							
60	YH 411 / HNB	*Indigofera pulchra* Willd.	Zounhô (Zuko), adoma (f);	Leguminosae	Tf	Dec	Oral + Topic	1.83	1.61	100	0	
61	YH 379 / HNB	*Jatropha gossypiifolia* L.	Nyikpotin, gbagidi kpotin (f, g);	Euphorbiaceae	Tf	Dec	Oral + Topic	6.42	5.65	29	71	
62	YH 380 / HNB	*Kedrostis foetidissima* (Jacq.) Cogn.	Jixo cyoma; Tchioma (f)	Cucurbitaceae	Fr	Pounding	Topic	0.92	0.81	0	100	
63	YH 381 / HNB	*Khaya senegalensis* (Desr.) A. Juss.	Zunzatin (f) / Agawu /Tere (g)	Meliaceae	B	Dec	Oral + Topic	20.18	17.74	91	9	
64	YH 382 / HNB	*Kigelia africana* (Lam.) Benth.	Nyablikpo (f);	Bignoniaceae	Tf	Dec; Mac	Oral + Topic	2.75	2.42	33	67	
65	AA 6749 / HNB	*Lantana camara* L.	Hla Ciyayo / Hla Coyo (f) /Zansoukpê man/ Hla Ciyamadidwe (G)	Verbenaceae	Tf	Dec; Pounding	Oral + Topic	26.61	23.39	90	10	
66	YH 384 / HNB	*Lawsonia inermis* L.	Lalitin (f); laritin (g); lali (y, n)	Lythraceae	Tf	Dec	Oral	0.92	0.81	100	0	
67	AA 6750 / HNB	*Lippia multiflora* Moldenke	Agala (f)/ Aklala (g)	Verbenaceae	Tf	Dec; Pounding	Oral + Topic	22.94	20.16	96	4	
68	YH 385 / HNB	*Mangifera indica* L.	Manga, amanga (f)	Anacardiaceae	B	Dec	Oral	4.59	4.03	80	20	
					R							
69	YH 386 / HNB	*Melaleuca leucadendra* (L.) L.	Kpenma sèmèton (f).	Myrtaceae	Tf	Dec	Oral	0.92	0.81	100	0	
70	YH 388 / HNB	*Mitracarpus hirtus* (L.) DC.	Godokwe, Godoko (f);	Rubiaceae	Pe	Pounding	Oral + Topic	4.59	4.03	100	0	
71	YH 389 / HNB	*Momordica charantia* L.	Nyensinken (f)	Cucurbitaceae	Pe	Dec; Mac	Oral + Topic	5.5	4.84	83	17	
72	YH 375 / HNB	*Mondia whitei* (Hook.f.) Skeels	Cirigun (f)	Apocynaceae	Tf	Pounding; Mac	Oral	0.92	0.81	100	0	
73	YH 319 / HNB	*Monodora myristica* (Gaertn.) Dunal	Sasalikun, sasagbakun (f,g)	Annonaceae	Fr	Dec; Mac; Grilling	Oral + Topic	14.68	12.9	75	25	
74	YH 320 / HNB	*Morinda lucida* Benth.	Xwensin	Rubiaceae	R	Dec	Oral	0.92	0.81	100	0	
75	YH 390 / HNB	*Musa × paradisiaca* L.	Kokwé azo	Musaceae	Le	Dec	Oral + Topic	1.83	1.61	100	0	
76	YH 391 / HNB	*Ocimum americanum* L.	Kesu kesu, xesu xesu, xisi xisi (f); akohun (g)	Lamiaceae	Pe	Dec; Pounding	Oral + Topic	17.43	15.32	84	16	
77	YH 325 / HNB	*Ocimum gratissimum* L.	Tchao, Ciyayo (f)	Lamiaceae	Tf	Dec; Pounding	Oral + Topic	22.94	20.16	68	32	[42]
78	YH 392 / HNB	*Olax subscorpioides* Oliv.	Mitin, mitun (f);	Olacaceae	R	Dec	Oral + Topic	2.75	2.42	67	33	
79	YH 393 / HNB	*Parkia biglobosa* (Jacq.) G Don	Ahwatin, afiti (f)	Leguminosae	B	Pounding	Oral + Topic	0.92	0.81	0	100	[58]
80	YH 394 / HNB	*Paullinia pinnata* L.	Xedulinifen (f, g);	Sapindaceae	Tf	Dec	Oral + Topic	4.59	4.03	100	0	
81	YH 397 / HNB	*Persicaria senegalensis* (Meisn.) Soják	Towe (g);	Polygonaceae	Tf	Dec	Oral + Topic	4.59	4.03	100	0	
82	YH 398 / HNB	*Philenoptera laxiflora* (Guill. & Perr.) Roberty,	Ahoma; Aho ma (f)	Leguminosae	Le	Pounding	Topic	0.92	0.81	0	100	
83	YH 387 / HNB	*Phymatosorus scolopendria* (Burm. f.) Pic. Serm.	Duma (f), Degoma (g).	Polypodiaceae	Tf	Dec	Oral + Topic	2.75	2.42	100	0	
84	YH 401 / HNB	*Piper nigrum* L.	Lènkun, (f); lènlènkun g)	Piperaceae	Fr	Dec	Oral	0.92	0.81	0	100	
85	YH 399 / HNB	*Pleiocarpa pycnantha* (K.Schum.) Stapf	Danyè (f); Vonma (g);	Apocynaceae	Tf	Dec	Oral + Topic	1.83	1.61	100	0	
86	YH 402 / HNB	*Pseudocedrela kotschyi* (Schweinf.) Harms	Atindodokpwe / Atinsudo dokpo (f)	Meliaceae	Tf	Dec	Oral + Topic	5.5	4.84	100	0	
					R							
87	AA 6753 / HNB	*Pteleopsis suberosa* Engl. & Diels	Kulu Kuli (f)/ Klwi-Klwi (f)	Combretaceae	B	Dec	Oral + Topic	29.36	25.81	94	6	[26, 59]
88	YH 403 / HNB	*Pterocarpus erinaceus* Poir.	Gbègbètin (f)	Leguminosae	B	Dec	Topic	0.92	0.81	0	100	
89	YH 404 / HNB	*Rhaphiostylis beninensis* (Hook.f. ex Planch.) Planch. ex Benth.	Kplakplama (f)	Icacinaceae	Tf	Dec	Oral + Topic	0.92	0.81	100	0	
90	YH 405 / HNB	*Ricinus communis* L.	Kasu wayi (a)	Euphorbiaceae	Tf	R.cend	Oral	1.83	0.81	100	0	
91	YH 261 / HNB	*Rourea coccinea* (Thonn. ex Schumach.) Benth.	Nociovijè, (f)	Connaraceae	R	Dec	Oral + Topic	1.83	1.61	100	0	
92	YH 406 / HNB	*Sarcocephalus latifolius* (Sm.) E. A. Bruce	Ko (ma) (f); kodo (g)	Rubiaceae	Tf	Dec	Oral + Topic	4.59	4.03	60	40	
					R							
93	YH 407 / HNB	*Schwenckia americana* L.	Amakwinkwin, zlon (f)	Solanaceae	Tf	Dec	Oral + Topic	8.26	7.26	100	0	
94	YH 364 / HNB	*Secamone afzelii* (Roem. & Schult.) K.Schum	Anonsima, zounkoudjou (f);	Apocynaceae	Fr	Dec	Oral	0.92	0.81	0	100	
95	YH 408 / HNB	*Securidaca longipedunculata* Fresen.	Kpata / Kpɛta (f)	Polygalaceae	R	Dec; Pounding	Oral + Topic	3.67	3.23	75	25	
96	YH 409 / HNB	*Senna alata* (L.) Roxb.	Amasu yovotòn (f);	Leguminosae	Tf	Dec; Pounding	Oral + Topic	5.5	4.84	67	33	
97	YH 413 / HNB	*Senna italica* Mill.	Agoè agoè, agogwè, adwe agwe (f).	Leguminosae	Tf	Dec; Pounding	Oral + Topic	1.83	1.61	100	0	
98	YH 410 / HNB	*Senna occidentalis* (L.) Link	Kinkéliba	Leguminosae	Tf	Dec	Oral + Topic	0.92	0.81	100	0	
99	YH 396 / HNB	*Spondias mombin* L.	Aklokontin/Ahlihon (f)	Anacardiaceae	R	Dec	Topic	0.92	0.81	100	0	
100	YH 412 / HNB	*Syzygium aromaticum* (L.) Merr. & Perr	Atinkɛn Gbadota (f)	Myrtaceae	Fr	Dec; Poudre	Oral + Topic	7.34	6.45	75	25	
101	YH 414 / HNB	*Terminalia glaucescens* Planch. ex Benth.	Alotun; Anagositin (f)	Combretaceae	R	Dec	Topic	0.92	0.81	100	0	
102	YH 415 / HNB	*Tetrapleura tetraptera* (Schumach. & Thonn.) Taub.	Lendja (f);	Leguminosae	Fr	Dec	Oral + Topic	4.59	4.03	100	0	
103	YH 416 / HNB	*Thalia geniculata* L.	Aflema (f).	Marantaceae	Tf	Dec; Mac	Oral	3.67	3.23	75	25	
104	YH 417 / HNB	*Tribulus terrestris* L.	Ahwanglon assou (f)/ Kpononmi (Mi).	Zygophyllaceae	Tf	Dec; Pounding	Oral + Topic	0.92	0.81	100	0	
105	AA 6751 / HNB	*Uvaria chamae* P. Beauv.	Aylaha / Ayadaxa/ Win Nyaxa, zinwokokwe, wianxa (f)/ Avun	Annonaceae	R	Dec	Oral + Topic	9.17	8.06	70	30	
106	YH 333 / HNB	*Xylopia aethiopica* (Dunal) A. Rich.	Kpéjélékun (f);	Annonaceae	Fr	Dec; Mac; Pounding	Oral + Topic	19.27	16.94	71	29	[39]
107	YH 334 / HNB	*Zea mays* L.	Gbadé (f);	Poaceae	Tf	Dec	Oral + Topic	0.92	0.81	100	0	
108	YH 335 / HNB	*Zingiber officinale* Roscoe	Dotè (f;g)	Zingiberaceae	Fr	Dec	Oral	0.92	0.81	100	0	
109	YH 422 / HNB	*Zornia glochidiata* DC.	Lèkun lèkun (f)	Leguminosae	R	Dec	Oral	0.92	0.81	100	0	
					B							

f: Fon; g: Goun; fr: french B: Bark; Le: Leaves; Tf: Leaf stem; Fr: Fruit; R: Root; Pe: Whole plant; L: Liana; S: Stem; Dec: Decoction; Mac: Mac: Maceration; IFh: Fidelity index for herborists; IFt: Fidelity index for traditional healers; RCend. To make ashes; Fc: Citation frequency

**Fig. 6:**
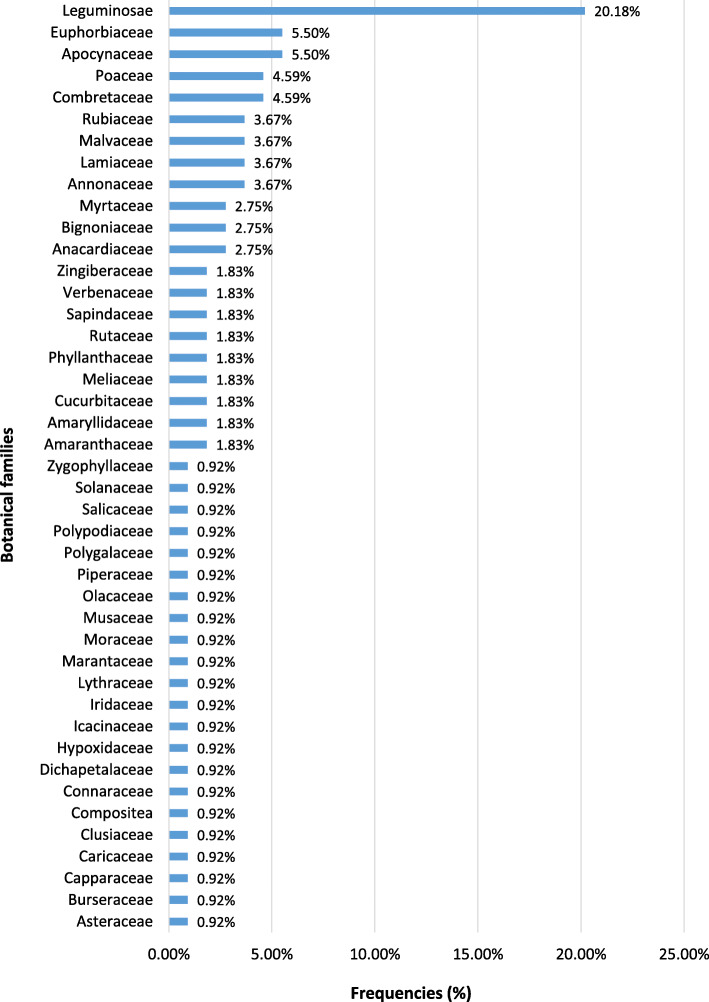
Distribution of botanical families

The most represented families were Leguminosae (20.18%) followed by Euphorbiaceae (5.50%), Apocynaceae (5.50%), poaceae (4.59%) and Combretaceae (4.59%) (Fig. 6).

The most frequently cited species were *Pteleopsis suberosa* Engl. & Diels, *Lantana camara* L., *Cyanthillium cinereum* (L.) H. Rob, *Ocimum gratissimum* L. and *Lippia multiflora* Moldenke with respectively 29.36, 26.61 and 22.94% citation frequencies respectively. The most frequently mentioned species did not necessarily belong in order to the most represented botanical families. Their respective frequencies of involvement in the composition of the recipes (Cpr) were 25.81, 23.39, 20.00, 20.00 and 20.00% respectively and their pairs of relative loyalty indices (herbalists/Traditional healers) were (93.75%; 6.25%), (89.66%; 10.34%), (100.00%; 00.00%), (68.00%; 32.00%) and (96.00%; 4.00%) respectively. Among these species, *Ocimum gratissimum* L. was the only plant strongly cited by both market herbalists (66,67%) and traditional healers (33,33%) and involved in about 20% of the recipes provided (Table 3). Also, it should be noted that even if *Cyanthillium cinereum* (L.) H. Rob and *Lippia multiflora* Moldenke seem to be two species widely used by herbalists, traditional healers did not use them so frequently.

In addition, seven (07) species of plants inventoried in this study are on the IUCN Red List as Near Threatened (*Cajanus cajan* (L.) Millsp., *Eucalyptus camaldulensis* Dehnh.); Vulnerable (*Afzelia Africana* Pers.*, Garcinia kola* Heckel*, Gossypium hirsutum* L.*, Khaya senegalensis* (Desr.) A. Juss.); and Endangered (*Pterocarpus erinaceus* Poir.).

**Fig. 7:**
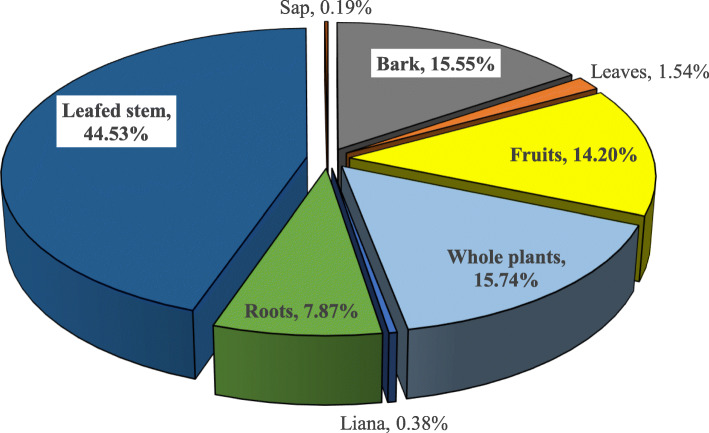
Plant parts involved in recipes

Different plant organs are used to treat different candidiasis. At the end of this survey, eight (08) plant organs were involved in the composition of traditional recipes (Fig. 7). Leafy stems (44.53%) were the most commonly used, followed by whole plants (15.74%) and bark (15.55%). Roots have only a minor role (7.87%) in recipes against candidiasis. Indeed, leafy stems and/or leaves are very important in medicinal recipes since they would constitute the basis for the synthesis of the majority of phytochemical compounds.

## Discussion

The knowledge of the plants used in the treatment of candidiasis is at the level of the elderly. Indeed, in Benin as elsewhere, endogenous knowledge is often hold by elder or wise people. Thus, properties of medicinal plants are ancestral knowledge that is only transmitted from one generation to another [60, 61]. Other surveys conducted in practitioners Africa on endogenous care practices yielded some findings comparable to ours. In addition, the high degree of seniority of traditional in this care counsellor has also been reported in surveys dealing with medicinal plants [50, 62]. In this sense, As Zougagh et al. [50], others authors argued that deep knowledge on the use of plants for healing purposes could only be acquired after years of practice [63, 64].

The female predominance of market herbalists and male predominance of traditional healers observed in this study could be explained by the fact that in Benin, sales at the market is an activity mainly carried out by women. Surveys on the traditional use of plants against infections carried out in Benin in the same geographical area support our results [42, 45, 65]. Similar findings were obtained in research activities carried out in other African area highlighting the female-biased sales from the age group of 40 years and over [64, 66]. However, in other parts of Africa, such as Morocco, the sale of medicinal plants is an activity exclusively done by men [50]. On the other hand, all the traditional healers in the current study were male, unlike the results reported by Klotoé et al. (2013) where both sexes were represented with a male predominance [44].

The responders (men/women) in our study were mostly illiterate. Our results concordance with those of other authors working in South Benin [44, 67, 68] with focus on the education rate among traditional healers (68.19%) compared to market herbalists (17.65%). But, unlike our study, Koudokpon et al. (2017) had only illiterate market herbalists [68]. Differences observed in our study could be due to the fact that our study dealt with almost the whole southern part of Benin and took into account more traditional healers than theirs, which was limited to two cities.

Regarding plant recipes, Klotoé et al. (2018) in their investigations of anti-hemorrhage plants found as in this study, that recipes based on medicinal plants provided by traditional healers contain mineral compounds [69]. The addition of mineral compounds to some compositions especially among traditional healers would have a stabilizing role.

Many studies showed that in practice, decoction was the most common method of preparing herbal recipes often indicated by traditional healers. Indeed, in Benin, the recent work of Koudokpon et al. (2017) on plants used in the treatment of infections and that of Fah et al. had reached the same conclusions [67, 68]. In other area in Africa, several authors in Togo, Nigeria, Congo and South Africa had also found that decoction was the most common preparation method used by traditional healers [62, 54, 70, 71]. Kinda et al. (2017) reports that this method is the most efficient way to extract bioactive compounds from plants [72]. This may explain why many traditional healers use it most often.

The oral administration of the preparations was the preparation method, the most recommended way of administration. According to many other authors, it is also the route of administration for most herbal preparations both in our study area [61, 67] and in other countries [70, 71, 73–75].

The very low degree of Informant Consensus Factor (ICF) in this study could be justified by the difference in the composition of the recipes served. The diversity of single species cited, often involved in the recipes provided, and could justify this weak consensus. This could also be related to socio-cultural factors. Since the populations of southern Benin are of different ethnic groups and cultures, endogenous practices regarding the use of medicinal plants could be different. Indeed, several ethnic groups were met during the survey. These include: Aïzo (Atlantic Department); Fons (Littoral and Zou Departments); Idaasha, Ifè, Isha (Collines Department); Mahi (Zou and Collines Departments); Goun, Yoruba (Ouémé and Plateau Department); Adja (Mono and Couffo Departments). Some authors who have worked in the same geographical area found the inhabitants belonging to different socio-cultural groups [76–78]. This study therefore shows that Benin is home for a wide variety of medicinal plant species used in the treatment of candidiasis.

The botanical families most cited in this work (Leguminosae, Euphorbiaceae, Apocynaceae and Combretaceae) were similar to those obtained by Koudokpon et al. (2017) who, in their studies on plants used in the treatment of infections pointing out that Leguminosae species were predominant in the recorded species [45, 79]; candidiasis being infections due to Candida yeasts. However, other researchers in Africa found species belonging the Fabaceae family the most represented [64, 70]. This could be related first to geographical conditions (nature of soils, climatic and other factors) that did not always favor the growth of the same plant species on different soil types and having then a significant influence on bioactive compounds, but also to socio-cultural factors [63]. Since knowledge on the therapeutic use of plants was often transmitted from one generation to another, the plants indicated in the treatment of a disease may also differ from one location to another or from one ethnic group to another.

Since few ethnobotanical surveys on candidiasis were specifically carried out, our results on the plant organs used was similar to those of many recent studies on plants with antimicrobial properties [68, 79]. However, unlike many plant studies, Kinda et al.(2017) found in an ethnobotanical survey of plants used in neuropsychiatric disorders that plant roots were the most commonly used by traditional healers [72].

The frequent involvement of leafy stems in recipes could be explained by the fact that the phytochemical compounds responsible for antifungal effects are more concentrated in these plant organs than others. Chemical groups are reported to be more abundant in this plant organs, where secondary metabolites are synthesized [79]. Castillo et al. reported that terpenes, tannins, flavonoids, essential oils, alkaloids, lecithin and polypeptides are the chemical compounds with antifungal properties in plants [80]. These properties observed with coriander essential oil on Candida spp. strains are reported by Freires et al. to be related to monoterpenes and sesquiterpenes present in the leaves of this plant [81]. In addition, ethnobotanical surveys conducted in Benin on medicinal plants sold in Benin have shown that leaf stems are the most commonly used plant organs by medicinal plant sellers, that decoction is the most recommended method of preparation by traditional practitioners and that the oral route is the most commonly used for the administration of medicinal plant recipes [57, 69]. Among plants identified in this survey, nine are previously cited. These ones are: Allium sativum L. [55]; Catharanthus roseus (L.) G.Don [31]; Cymbopogon citratus (DC.) Stapf (79); Garcinia kola Heckel [55]; Hyptis suaveolens (L.) Poit [30].; Ocimum gratissimum L. [42], Parkia biglobosa (Jacq.) G Don [58]; Pteleopsis suberosa Engl. & Diels [26, 59]; Xylopia aethiopica (Dunal) A. Rich [39].

Among the 109 medicinal plants species identified in the treatment of candidiasis in southern Benin, 7 species are listed as near threatened plants on the IUCN red list. This confirms the anthropogenic pressure exerted on plant resources and raises the question of plant conservation. Indeed, as shown by Djégo et al. (2011), in Benin, deforestation leads to the disappearance of several medicinal plants [42]. It is therefore important to sensitize the populations on the conservation of plant biodiversity in order to guarantee access to medicinal plants for future generations.

## Conclusion

This current ethnobotanical study showed that South Benin is an overflowing area with many species of medicinal plants indicated the traditional treatment of candidiasis. Market herbalists and traditional healers have indicated 109 medicinal plants in the treatment of candidiasis. The most used species are *P. suberosa*, *L. camara*, *C. cinereum*, *O. gratissimum* and *L. multiflora*.

The various species identified could be new sources of bioactive molecules. However, this requires further pharmacological and toxicological studies.

This study could be very useful to scientists for further research works in order to investigate experimentally the properties of the plant species thus identified to effectively inhibit or even kill *Candida* strains involved in candidiasis. It could also be extended to the whole country in order to have a single database of medicinal plants used in the treatment of candidiasis.

## Supplementary information

**Additional file 1.**

## Data Availability

The datasets used and/or analysed during the current study available from the corresponding author on reasonable request.
