# Supplementary material for: Ethnobotanical survey on plants used in the treatment of candidiasis in traditional markets of southern Benin
Source: BMC Complement Med Ther. 2020 Sep 21;20:288. doi: 10.1186/s12906-020-03080-6 (PMC7507638; doi:10.1186/s12906-020-03080-6)
Supplement: Supplementary file 1 — Additional file 1. [file 12906_2020_3080_MOESM1_ESM.docx]

**ETHNOBOTANICAL SURVEY ON PLANTS USED IN THE TREATMENT OF CANDIDIASIS IN THE SOUTHERN BENIN**

QUESTIONNAIRE

Identification number :…………………………………….. Date ………./………./201 …

1. **Informant profile**

Q 1. Name :……………………………………………………………………………….

Q 2. Target [ ] : 1 = Traditional healer 2 = Herbalist

Q 3. Language of l’interview : ………………………………

Q 4. City ………………

Q 5. District ………………………………………

Q 6. Village……………

Q 7. Sex [ ] : 1 = Male 2 = Female

Q 8. Age : [ ] years

Q 9. Level of study  [ ]: 1 = Illeterate 2= Primary 3 = Secondary 4 = University

Q 10. Access to the practice of traditional medicine [ ] : 1 = by inheritance 2 = by training

3 = on-the-job training

Q 11. Professionl experience [ ] years

1. **Recipe Information**

Q 12. Type of candidiasis: [ ] 1 = skin 2 = genital 3 = buccal 4 = anal

Q 13. Number of plants in recipe [ ]

Q 14. Non-vegetable element in recipe [ ] : 1 = no 2 = yes ………………………………

Q 15. Method of preparation  [ ] : 1 = maceration 2 = infusion 3 = decoction

4 = trituration 6 = other…………………

Q 16. Route of administration  [ ] : 1 = oral 2 = local application 7 = other………….

Q 17. Duration of treatment : ………………………………………………….

Q 18. Prohibited during treatment ……………………………………………………………………

Q 19. Side effects …….………………………………………………………..................... …………………………………………………………………………………………………………

Q 20. Recipe price ……………………………………………………………………

1. **Information on the plants making up the recipe**

| Q 21.  Plants | Q 22.  Local name | Q 23.  Scientifique name | Q 24.  Part used^a^ | Q 25.  State of freshness^b^ | Q 26.  Collection method^c^ |
| --- | --- | --- | --- | --- | --- |
| Plant 1 |  |  |  |  |  |
| Plant 2 |  |  |  |  |  |
| Plant 3 |  |  |  |  |  |
| Plant 4 |  |  |  |  |  |
| Plant 5 |  |  |  |  |  |
| Plant 6 |  |  |  |  |  |
| Plant 7 |  |  |  |  |  |
| Plant 8 |  |  |  |  |  |
| Plant 9 |  |  |  |  |  |
| Plant 10 |  |  |  |  |  |

^a^ 1 = Whole plant ; 2 = leaf ; 3 = leafy stem ; 4 = root ; 5 = stem ; 6 = bark 7 = other…………………..

^b^ 1 = fresh 2 = dried 3 = other…………………

^c^: 1 = uprooting of whole plant 2 = vital organ ; 3 = renewable organ
